# Acceptance and utilization efficiency of a purple durum wheat genotype by *Sitophilus granarius* (L.)

**DOI:** 10.1038/s41598-023-41384-y

**Published:** 2023-08-30

**Authors:** Ilaria D’Isita, Antonella Marta Di Palma, Pasquale De Vita, Giacinto Salvatore Germinara

**Affiliations:** 1 Department of Agricultural Sciences, Food, Natural Resources and Engineering (DAFNE), University of Foggia, Via Napoli 25, 71122 Foggia, Italy; 2 CREA Research Centre for Cereal and Industrial Crops, 71122 Foggia, Italy

**Keywords:** Ecology, Plant sciences

## Abstract

The granary weevil (*Sitophilus granarius* L.) is a major primary pest of stored cereals throughout the world. Among the major classes of plant secondary metabolites, flavonoids can affect insect feeding behaviour and their growth rate. In this study, the susceptibility of an anthocyanin-rich purple durum wheat genotype (T1303) to the granary weevil was evaluated in comparison with two yellow durum (Ofanto) and bread (Mec) wheat varieties. The feeding response and food utilisation efficiency by adult insects was also investigated by calculating nutritional indices in whole flour disk bioassays. Different levels of susceptibility to granary weevil emerged among genotypes tested. The mean food consumption by an insect, F1 progeny, and female parental offspring calculated for the T1303 genotype were significantly lower than those of yellow kernel wheat varieties. Moreover, T1303 genotype induced deterrence in the adult insects as demonstrated by the positive values of the food deterrence index. Besides, relative grow rate and efficiency conversion of ingested food indices were negative for T1303 and positive for both yellow wheat varieties indicating respectively a decrease and an increase of insect body weight during the bioassays. Finally, a higher mortality rate was recorded for insects fed on T1303 flour disks compared to disks obtained from yellow wheat varieties. These results provide evidence for the antifeedant and toxic effects of anthocyanins present in the T1303 pericarp against the granary weevil. Overall, this study contributes new insights into the mechanisms of host acceptance and food utilization by *S. granarius* and would be useful to identify antifeedant flavonoids as well as to develop varietal resistance-based strategies against this pest.

## Introduction

Wheat is one of the most important food crops to human populations consumed worldwide^1^. Food security will depend on the ability to increase productivity while limiting losses during both cultivation^2^ and postharvest^3^. Insect pests during product storage are an increasingly global problem^4^. In fact, postharvest wheat losses, due to pest attacks, are estimated to be about 10–15% of the global annual production^5,6^ and, in some developing countries, they can reach 50% of the total harvest^7^.

The granary weevil, *Sitophilus granarius* (L.) (Coleoptera, Curculionidae), is one of the most damaging primary pests of stored cereals worldwide. It can attack intact kernels and causes both severe quantitative and qualitative losses^5,8–10^ due to larvae and adults feeding and commodities contamination with exuviae, excrements and mycotoxins that may result from insect-promoted fungal growth during storage^5,8–13^.

The endophytic development of immature stages, the stringent legislation on the use of synthetic pesticides and the increasing consumer demand for safer food make the control of granary weevil very difficult^4,14^. Thus, sustainable control means as alternatives to chemical inputs during cereal storage are urgently needed^13,15^. Possible alternative control methods include the use of botanicals powders, extracts, and essential oils (EOs) of plant origin^13,16–23^, semiochemicals^12,14,24^, inert powder^25–31^ and resistant varieties^32^.

Food plant selection by phytophagous insects consists of food finding and food acceptance^33^. Volatile organic compounds (VOCs) play an important role in host finding because they are the first chemicals detected and used by insects to distinguish between suitable habitats and substrates and unsuitable ones^34–37^. The host-plant acceptance mainly depends on behavioural responses of insects to non-volatile plant chemical and physical features^37^.

*Sitophilus granarius* adults are able to perceive a wide range of volatile compounds^38^ emitted by grains of several cereals^39,40^ and are attracted to the odour blend of commercial wheat with yellow kernels^41,42^. Moreover, plant secondary metabolites are involved in insect-plant interactions from habitat selection to host acceptance^37,43–45^.

Major class of secondary metabolites are flavonoids, including anthocyanins, which represent about 5–10% of the known secondary products in plants^46^ and are significant cues in host recognition and host acceptance^43,44,47^.

Several studies showed the dual nature of flavonoids as insect feeding stimulants or deterrents. Hamamura et al*.*^48^ found sitosterol and the flavonol isoquercitrin as biting factors for the silkworm, *Bombyx mori* L. Moreover, flavonoids could have insect feeding stimulant activity^49–53^. By contrast, many flavonoids are deterrents to insects, may be deleterious if ingested and detrimental to their growth^37,44^. Flavonoids have also been implicated as plant compounds that stimulate or deter the female oviposition^43,44^.

The increasing interest in the health benefits of flavonoids, has prompted plant breeders to increment the levels of these compounds in crops^54^. In this context, pigmented wheat genotypes that carry purple genes controlling anthocyanin pigmentation on grain pericarp are very interesting^55–57^, due to the high nutritional value conferred to the final products^57–59^.

In a previous study, marked differences in VOCs profile emerged within pigmented and yellow kernel of durum and bread wheat genotypes and adults of *S. granarius* did not exhibit a preferential orientation toward the odours of pigmented wheat genotypes during behavioural bioassays^60^.

The aim of the present study was to investigate the influence of wheat anthocyanin pigments in host acceptance and utilization by *S. granarius* adults. To this end, intact kernel susceptibility tests and flour disks bioassays were carried out with two commercial yellow wheat varieties and a pigmented wheat genotype with a purple pericarp.

## Materials and methods

### Insects

*Sitophilus granarius* were reared for several generations on whole durum wheat kernels (var. Simeto) in cylindrical glass containers (Ø 15 × 15 cm) covered with a fine mesh net (0.5 mm). Colonies were maintained in the dark at 25 ± 2 °C and 60 ± 5% relative humidity. Mixed sexes 30-day-old adult beetles were used for intact kernel susceptibility tests and flour disks bioassays.

### Plant materials and grain quality assessment

Three wheat genotypes were chosen for this study, including the bread wheat (*Triticum aestivum* L. subsp. *aestivum*) variety “Mec” (Marzotto/Combine) and two durum wheat [(*Triticum turgidum* L. subsp. *durum* (Desf.)] genotypes: “Ofanto” (Adamello/Appulo), an élite variety with high grain yield and wide adaptability to the Mediterranean basin^61^, and a purple durum wheat genotype, “T1303” (USDA code PI 352395) with high levels of anthocyanins in the grain. The three wheat genotypes were grown simultaneously in a replicated (n = 10) field trial carried out at the CREA-CI of Foggia, Italy (41°28′ N, 15°32′ E; 75 m a.s.l.), on a clay-loam soil (Typic Chromoxerert) during the 2019–2020 growing season, using standard agronomic practices. The harvested wheat genotypes were analysed to determine the main qualitative and technological parameters and stored at low temperature (4 ± 1 °C) until needed for biological tests. Moisture of whole grains was determined using the single-stage air oven (Termostabil k3, Cavallo S.R.L., Milan, Italy) method (ASAE 2003). This method utilizes whole grain dried for 18 h at 130 °C to determine the moisture content. Thousand-kernel weight (TKW) was calculated from the mean weight of three sets of 500 grains per each wheat genotype. Protein content (PC) was determined by nitrogen combustion analysis according to Approved Method 46–30 (AACC International 2010) using a Dumas nitrogen analyzer (Leco Corp, St. Joseph, MI). The Single Kernel Characterization System 4100 (SKCS) (Perten Instruments, North America, Inc., Springfield, IL, USA) was used to characterize kernels hardness (Ha) using a sample of 300 kernels (Method 55-31) (AACC, 2010). A grain sample with 800 g of each sample were also milled by a Bona 4RB mill (Bona, Monza, Italy) after tempering according to their hardness. The flour obtained was used to perform alveograph test, according to ICC-Standard no. 121 (ICC, 1992). The variables of alveograph deformation energy (W) and curve configuration ratio [P/L-relation between dough tenacity (P) and extensibility (L)] were measured.

Total anthocyanin content (TAC) was evaluated using a colorimetric method with different pH solutions as reported by Ficco et al.^57^. Briefly, two aliquots of the supernatants extracted (750 μL) were put into different tubes and diluted (1:2, v/v) with either potassium chloride buffer (0.03 M KCl), for pH 1.00, or sodium acetate buffer (0.4 M CH_3_CO_2_Na·3H_2_O), for pH 4.50. The resulting samples were incubated for 30 min at room temperature in the dark and then filtered with 0.45 μm regenerated cellulose syringe filters. The absorbances of the samples at 520 nm were measured against distilled water as the blank. Total anthocyanin content was corrected for the dry matter and is expressed as Cy-3-Glc equivalents as micrograms per gram of dry matter.

In order to ensure the absence of live insects inside the wheat kernels to be used for biological bioassays, samples were frozen at − 20 °C for 72 h before the experiments.

### Susceptibility tests

Susceptibility tests with intact kernels were performed on wheat samples conditioned for 7 days at 25 ± 2 °C, 60 ± 5% r.h. after frozen treatment. For each wheat genotype, not infested kernel samples (60 g) placed in cylindrical glass containers (Ø 9 × 14.5 cm) were infested with 12 *S. granarius* adults of mixed sexes. Containers were closed by screw caps and maintained in the incubator (Memmert GmbH + Co. KG IN 110 plus, Schwabach, Germania) at controlled conditions of photoperiod (L 0 : D 24), temperature (25 ± 2 °C), and relative humidity (60 ± 5%). For each wheat genotype there were 5 replicates. After 15 days exposure, insects were removed, sexed and the number of dead insects in each replicate was recorded. The F1 progeny was monitored by removing and counting newly emerged adults every 3 days. The experiment was terminated when no adults emerged for five consecutive days^62^.

For each wheat genotype, the following parameters were calculated: (1) total number of F1 progeny; (2) median development period (D), estimated as the time, expressed in days, from the middle of the oviposition period to the emergence of 50% of the F1 generation^63^; (3) percentage of mortality during the oviposition period; (4) number of adult offspring per female; (5) percentage of weight loss = Wi–Wf/Wi × 100 were Wi = Initial dry weight and Wf = Final dry weight^64^; (6) food consumption by an insect.

### Flour disks bioassays

For each genotype, whole wheat flour was prepared by milling kernel samples (20 g) using a Tecator Cyclotec 1093 (International PBI, Milano, Italy) laboratory mill (1 mm screen-60 mesh). A sample (2.5 g) of each wheat flour was uniformly suspended in distilled water (8 mL) and stirred by a magnetic stirrer (MS-H280-PRO, DLAB Scientific Co., Beijing, China).

To obtain flour disks to be used in feeding bioassays, aliquots (200 μL) of suspension were dropped onto holes (Ø 1 cm, height 3 mm) of a rectangular support (15 cm × 15 cm) designed for this purpose and manufactured using a 3D printer (Zortrax S.A., Olsztyn, Poland). Then, the support was placed in a fume cupboard for 7 h until solid flour disks were obtained. Initial humidity of flour disks was stabilized overnight at 25 ± 2 °C in an airtight glass desiccator using a NaCl solution^65^ that generated 60 ± 5% r.h.

In a pre-weighed glass vial (22 mL) two flour disks and 5 group-weighed weevil adults were introduced. Each vial was then re-weighed using an analytical balance (AS R2 PLUS series, Radwag Headquarters, Radom, Poland) and maintained in the incubator (Memmert GmbH + Co. KG IN 110 plus, Schwabach, Germania) at controlled conditions of photoperiod (L 0 : D 24), temperature (25 ± 2 °C), and relative humidity (60 ± 5%) for 6 days. For each genotype 5 replicates were set up. After 6 days, the glass vials with flour disks and alive insects were weighed again and the number of dead insects was recorded.

Six days after the experiment start, the adult weevil mortality rate (%) was calculated. Moreover, the following nutritional indices were calculated: Relative Consumption Rate (RCR) = D/(B × day), where D = biomass ingested (mg)/No. of live insects on the sixth day; Relative Growth Rate (RGR) = (A − B)/(B × day), where A = mean weight (mg) of live insects on the sixth day, B = initial mean weight (mg) of insects; Efficiency Conversion of Ingested Food (ECI) = (RGR/RCR) × 100; Feeding Deterrence Index (FDI) (%) = [(C − T)/C] × 100, where C = consumption of control disks (Mec or Ofanto) and T = consumption of disks of the genotype considered (T1303)^66,67^.

### Statistical analysis

Data were submitted to analysis of variance (ANOVA) followed by Tukey’s HSD test for mean comparisons. Before ANOVA, data were submitted to Shapiro–Wilk’s test to verify the normal distribution of data and to Levene’s test to assess the homogeneity of variances. Statistical analyses were performed with SPSS (Statistical Package for the Social Sciences) v.18 for Windows (SPSS Inc., Chicago, IL).

### Plants materials

Plant materials used in the present study are compliant with the local and national guidelines.

### Ethics approval

This article does not contain any studies with human participants or animals performed by any of the authors.

### Informed consent

Informed consent was obtained from all individual participants included in the study.

## Results

### Qualitative and technological parameters

The average values of the main qualitative and technological parameters determined on the wheat kernel samples immediately after harvesting are reported in Table 1. The average moisture values were the same for all the three wheat genotypes analyzed, while the other parameters showed statistically significant differences (TKW: F = 8.254 *df* = 2 *P* = 0.019; PC: F = 19.397 *df* = 2 *P* = 0.002; Ha: F = 3309.80 *df* = 2 *P* = 0.000; W: F = 1393.00 *df* = 2 *P* = 0.000; P/L: F = 28.231 *df* = 2 *P* = 0.000). The commercial durum wheat variety (Ofanto) showed the highest TKW and Ha values but its PC (13.5 ± 0.5%) was significantly lower than those of the other genotypes. The bread wheat variety Mec showed the lowest values of Ha, being a variety with medium/soft kernels, and the most suitable alveographic parameters for making bread (high value of W and low of P/L). The T1303 genotype was the only one with the presence of anthocyanins in the grain (40.2 ± 0.03 mg/kg), a high protein content (15.4 ± 0.8%), similar to Mec (16.1 ± 0.5%), and a Ha value (85 ± 3) similar to that of the Ofanto variety (88 ± 3).

**Table 1:** Phenotypic characterization of the yellow varieties (Mec, Ofanto) and the pigmented genotype (T1303) evaluated in this study.

Genotype (kernel color)	Moisture (%) (Mean ± S.E.)	TKW^a^ (g) (Mean ± S.E.)	PC^b^ (% d.m.) (Mean ± S.E.)	Ha^c^	TAC^d^ (mg/kg) (Mean ± S.E.)	W-value^e^ (10^−4^ Joule) (Mean ± S.E.)	P/L^f^ (%) (Mean ± S.E.)
Bread wheat							
Mec (yellow)	10.3 ± 0.4 a	40.0 ± 1.2 a	16.1 ± 0.5 b	M = 44 ± 4 a	nd	190 ± 8 c	0.6 ± 0.02 a
Durum wheat							
Ofanto (yellow)	9.6 ± 0.4 a	46.0 ± 1.3 b	13.5 ± 0.5 a	H = 88 ± 3 b	nd	92 ± 6 b	1.2 ± 0.03 b
T1303 (purple)	10.4 ± 0.3 a	41.0 ± 1.5 a	15.4 ± 0.8 b	H = 85 ± 3 b	40.2 ± 0.03	45 ± 5 a	0.9 ± 0.04 ab

In the same column, values with different letters are significantly different at *P* < 0.05.

^a^Thousand-kernel weight. ^b^Protein content. ^c^Kernels hardness. ^d^Total anthocyanin content. ^e^Alveograph deformation energy. ^f^Curve configuration ratio.

### Susceptibility tests

During the oviposition period, the mean mortality rate of *S. granarius* adults kept on the purple kernel genotype T1303 (16.7 ± 4.8%) was higher than those observed for the yellow wheat varieties, even if not significantly different (*F* = 0.700; *df* = 2; *P* = 0.533) (Table 2).

**Table 2:** Percentage of mortality during the oviposition period, number of F1 progeny, offspring produced by a parental female and the median development period of *S. granarius* adults reared on yellow varieties (Mec, Ofanto) and pigmented genotype (T1303).

Genotype (kernel color)	Adult mortality (%) (Mean ± S.E.)	F1 progeny (n.) (Mean ± S.E.)	Offspring/female (n.) (Mean ± S.E.)	Development period (days) (Mean ± S.E.)
Bread wheat				
Mec (yellow)	8.33** ± **4.81 a	213.67** ± **26.99 b	44.02** ± **4.23 b	40.99** ± **0.57 a
Durum wheat				
Ofanto (yellow)	11.11** ± **5.56 a	213.00** ± **6.11 b	47.67** ± **7.09 b	40.68** ± **0.41a
T1303 (purple)	16.66** ± **4.81 a	67.00** ± **11.93 a	10.47** ± **0.39 a	42.12** ± **0.67 a

In the same column, values with different letters are significantly different at *P* < 0.05.

The number of F1 progeny emerged from the different genotypes showed significant differences (*F* = 23.572; *df* = 2; *P* = 0.001). The mean number of adults emerged from T1303 (67.0 ± 11.9) kernel samples was significantly lower than those obtained from Mec (213.7 ± 27.0) (*P* < 0.05; Tukey test) and Ofanto (213.0 ± 6.1) (*P* < 0.05; Tukey test) samples. This resulted in significant differences (*F* = 18.455; *df* = 2; *P* = 0.003) among the offspring originated by one parental female on different wheat genotypes (Table 2). By contrast, the median development period of *S. granarius* emerged from the purple wheat genotype T1303 (42.1 ± 0.7) was the highest but not significantly different (*F* = 1.767; *df* = 2; *P* = 0.249) compared to the yellow Mec (41.0 ± 0.6) and Ofanto (40.7 ± 0.4) varieties (Table 2).

The mean percentage of weight loss after the F1 emergence, corrected for changes in moisture content, showed significant differences (*F* = 150.272; *df* = 2; *P* < 0.001) among different genotypes (Table 3). The mean percentage of weight loss recorded for T1303 (1.46 ± 0.31%) was significantly lower (*P* < 0.05) than those observed for Mec (6.75 ± 0.31%) and Ofanto (7.02 ± 0.04%) varieties (Table 3). As a consequence, the mean food consumption by an insect emerged from the T1303 genotype (12.97 ± 0.44 mg) was significantly lower (*F* = 13.835; *df* = 2; *P* = 0.006) (*P* < 0.05; Tukey test) than those recorded for Mec (19.37 ± 1.57 mg) and Ofanto (19.82 ± 0.71 mg) varieties (Table 3).

**Table 3:** Damage by *S. granarius* on yellow (Mec, Ofanto) and pigmented (T1303) wheat genotypes.

Genotype (kernel color)	Final weight (mg) (Mean ± S.E.)	Weight loss % (Mean ± S.E.)	Food consumption by an insect (mg) (Mean ± S.E.)
Bread wheat			
Mec (yellow)	56.01** ± **0.19 a	6.75** ± **0.31 b	19.37** ± **1.57 b
Durum wheat			
Ofanto (yellow)	55.84** ± **0.04 a	7.02** ± **0.04 b	19.82** ± **0.71 b
T1303 (purple)	59.18** ± **0.21 b	1.46** ± **0.31 a	12.97** ± **0.44 a

In the same column, values with different letters are significantly different at *P* < 0.05.

### Flour disk bioassays

The mean RCR value of insects fed with flour disks obtained from the purple wheat genotype T1303 (0.057 ± 0.004 mg/mg/day) was lower than that of insects fed on disks of yellow wheat varieties, even if not significantly different (*F* = 1.095; *df* = 2; *P* = 0.366) (Table 4).

**Table 4:** Mortality, feeding deterrent index (FDI), relative consumption rate (RCR), relative growth rate (RGR), and efficiency conversion of ingested food (ECI) of *S. granarius* adults fed for six days on flour disks obtained from yellow bread (Mec) and durum (Ofanto) wheat varieties and pigmented wheat genotype (T1303).

Genotype (kernel color)	Mortality (%) (mean ± S.E.)	FDI (%) (mean ± S.E.)		RCR (mg/mg/day) (mean ± S.E.)	RGR (mg/mg/day) (mean ± S.E.)	ECI (%) (mean ± S.E.)
		Mec	Ofanto			
Bread wheat						
Mec (yellow)	0 a	–		0.065 ± 0.002 a	0.020** ± **0.003 b	30.7** ± **4.2 b
Durum wheat						
Ofanto (yellow)	0 a		–	0.062** ± **0.005 a	0.023** ± **0.005 b	36.9 ± 6.2 b
T1303 (purple)	24.0 ± 4.0 b	39.7 ± 4.6	32.2 ± 5.1	0.057** ± **0.004 a	− 0.025** ± **0.004 a	− 44.7** ± **6.2 a

In the same column, values with different letters are significantly different at *P* < 0.05.

Significant differences were found among the mean RGR and ECI indices calculated for different wheat genotypes (*F* = 43.943; *df* = 2; *P* < 0.01; *F* = 65.863; *df* = 2; *P* < 0.01, respectively). The mean RGR on flour disks from the T1303 purple genotype was negative (− 0.025 ± 0.004 mg/mg/day) and significantly lower (*P* < 0.05; Tukey test) than the positive RGR calculated for Mec and Ofanto (0.020 ± 0.003 and 0.023 ± 0.005 mg/mg/day, respectively) varieties (Table 4), indicating respectively a significant decrease and increase of the insect body weight during the experiment. As a consequence, the value of ECI index of T1303 flour disks (− 44.7 ± 6.2) was negative and significantly lower (*P* < 0.05; Tukey tests) than those of Mec (30.7 ± 4.2) and Ofanto (36.9 ± 6.2) flour disks (Table 4). Positive FDI indices were calculated for T1303 flour disks using the flour disk consumption of Mec (39.72 ± 4.60) or Ofanto (32.20 ± 5.12) as the control, indicating feeding deterrence (Table 4).

Six days after the experiment start, no mortality was observed for insects fed on Mec and Ofanto flour disks whereas a significant (*F* = 36.000; *df* = 2; *P* < 0.001) (P = 0.05; Tukey test) mean mortality rate (24.0 ± 4.0) was induced in insects fed on T1303 flour disks (Table 4).

## Discussion

Development of resistant wheat varieties to insect attacks during wheat grain storage is one of the most promising low-impact alternatives to insecticides in the management of stored grain pests^32^. Therefore, several studies aimed to develop antifeedant-based control means^44^ as well as to identify new possible sources of resistance to the stored-product pests useful in breeding programs^68–71^. For instance, the results of bioassay using transgenic wheat plants, containing a modified avidin gene, challenged with granary weevil revealed 100% mortality of the insects showing high levels of resistance^72^.

Pigmented wheat genotypes are characterized by a high antioxidant activity as well as large variations in the quality and composition of anthocyanins, that imparts purple, red or blue pigmentation in wheat^57^. Anthocyanins are involved in different kind of animal-plant interactions, which include the attraction of pollinators and frugivores and the repellence of herbivores and parasites^73^.

The interest in the anthocyanins content of pigmented cereals has increased due to their benefit on the human health as nutraceutical ingredients and functional foods^74–76^.

Early studies showed that polyphenol-rich pericarp purple corn extracts, containing anthocyanins, have several negative effects on the growth, development and fitness of different stages of *Manduca sexta* (L.) and *Spodoptera frugiperda* (JE Smith), suggesting their suitability as biopesticides^77–79^. By contrast, another study suggested that there are no differences in susceptibility to insect attacks during storage between white and red wheat varieties^80^.

In the present study, susceptibility bioassays revealed a significant reduction in the total number of F1 progeny and female parental offspring emerged from purple wheat genotype compared with yellow wheat varieties. Moreover, insects fed on pigmented grains consumed less food substrate than those reared on yellow varieties, which resulted in a significantly lower damage. These results strongly suggested a different level of susceptibility among the purple genotype and yellow varieties studied.

It is known that host acceptance by stored cereal pests depends on both physical and chemical properties of grain kernels. Indeed, the susceptibility of various wheat cultivars to insect pests has been related to physical kernel features, such as water content, hardness and diameter of kernel, thousand-kernel weight, vitreosity^81,82^ and content of some chemicals, such as protein or gluten, total lipids and cuticular lipids^83–88^ that are function of genetic and environmental factors^89,90^. Besides, the role of pericarp cell wall components in cereal weevil resistance has also been one of the aspects studied in the past on various cereals^91,92^. However, several studies have shown that the different varietal response to external stress was much more likely caused by complex interactions between structural factors (proteins and polysaccharides) and phytochemicals present in the grain^93–96^.

In our study, anthocyanins were contained exclusively in the pigmented genotype T1303 whereas qualitative and technological parameters of T1303 were similar to those of at least one of the two yellow wheat varieties. In fact, the pigmented T1303 genotype showed a thousand-kernel weight value and protein content similar to the yellow Mec variety and hardness values similar to the Ofanto variety.

In this contest, marked differences registered in population dynamics of *S. granarius* among yellow and purple kernel genotypes appeared to be associated with differences in the content of phenolic compounds, particularly anthocyanins. This is consistent with Kordan et al*.*^84^, which showed that a greater presence of phenolics in wheat grain determines a higher adult mortality and a reduction of insect fitness and damage.

Thus, considering the possible role of pericarp anthocyanins in the susceptibility of T1303, Mec and Ofanto to *S. granarius*, flour disk bioassays, using whole flour of each genotype, were set up to definitively overcome the influence of physical features.

However, although small differences in the amounts of flour disk ingested, different food conversion efficiency were found among the purple and yellow genotypes. Indeed, RGR and ECI values were positive for the Mec and Ofanto varieties and negative for T1303 indicating respectively an increase and a decrease of insect body weight during the experiments. Besides, the positive FDI values calculated for T1303 using Mec or Ofanto as controls indicated actual feeding deterrence of the T1303 genotype. Lastly, after six-day exposure, the mortality percentages of *S. granarius* adults fed on T1303 wheat flour disks were significantly higher than those observed for insects fed on yellow wheat varieties.

On the whole, our results strongly suggested that the anthocyanins accumulated in the pericarp of T1303 kernels, evenly distributed in the whole flour used for flour disks preparation, determined antifeedant, deterrent and toxic effects against granary weevil adults that may explain the differences in susceptibility observed. Certainly, a more thorough investigation will have to be conducted on other plant’ secondary metabolites that could interfere with the metabolism of the insect^96^.

In this context, our results pave the way to better understand the biological activity of the phenolic fraction of T1303 pericarp extracts and to identify their bioactive components. Finally, flour disk bioassays appear very promising for further wheat genetic investigation because they could be well suited to high-throughput analyses required by -omics approaches and, to accelerate the transfer of genes or genetic regions associated with resistance in a modern breeding program through marker-assisted selection. From a practical point of view, our results strongly suggest that purple wheat genotypes could be exploited in breeding programs to improve wheat resistance to the attacks of post-harvest stored pests, contributing to the alternative control options.

## Data Availability

All data generated or analysed during this study are included in this published article.
